# New Potential Antitumor Pyrazole Derivatives: Synthesis and Cytotoxic Evaluation

**DOI:** 10.3390/ijms141121805

**Published:** 2013-11-04

**Authors:** George Mihai Nitulescu, Constantin Draghici, Octavian Tudorel Olaru

**Affiliations:** 1 Faculty of Pharmacy, “Carol Davila” University of Medicine and Pharmacy, Traian Vuia 6, Bucharest 020956, Romania; E-Mail: octav_olaru2002@yahoo.com; 2 Organic Chemistry Center of the Romanian Academy “Costin D. Nenitescu”, Splaiul Independentei, 202B, Bucharest 060023, Romania; E-Mail: cst_drag@yahoo.com

**Keywords:** *Artemia salina*, *Daphnia magna*, *Triticum aestivum*, pyrazolyl thiourea

## Abstract

New pyrazole derivatives were designed and synthesized as potential protein kinase inhibitors in the view to develop specific antitumor therapies. The structures of the compounds were elucidated using spectral and elemental analyses. The antitumor potential was estimated using wheat seeds and the general toxicity was evaluated by alternative methods, using invertebrate animals. One 3-aminopyrazole derivative emerged as a potential candidate for the development of future cytotoxic compounds.

## 1. Introduction

The identification of the central role of protein kinases in cell signalling and their implication in malignant pathologies has led to extensive efforts to develop specific protein kinases inhibitors as treatment for a wide range of cancers [1]. Aminopyrazoles emerged as a powerful pharmacophore scaffold and they have been extensively used to design various kinase inhibitors.

Tozasertib, also known as VX-680, or MK-0457, is a 3-aminopyrazole derivative that inhibits Aurora kinases, thus inducing apoptosis in tumor cells, and was developed by structural optimization of an aminopyrazole quinazoline derivative [2]. ENMD-2076 is an orally-active analogue of tozasertib, based on the same 3-aminopyrazole template, and a potent inhibitor of Aurora A kinase and of other cancer-related kinases. ENMD-2076 has demonstrated significant preclinical activity and is tested in multiple clinical studies in order to develop specific antitumor therapies [3]. Barasertib is also an anticancer 3-aminopyrazole derivative acting through Aurora B kinase inhibition [4].

AT9283 is a multitargeted kinase inhibitor with potent Aurora kinase activity based on 4-pyrazolamine and urea pharmacophores [5]. The carbonyl group bound directly to the pyrazolamine proved to be involved in the interaction with the ATP-binding site in various kinases, making the pyrazolyl amide a powerful and versatile template in the design of kinase inhibitors. The usefulness of this scaffold can be observed in the structure of AT7519, a multi-cyclin-dependent kinase inhibitor with a good activity against a range of human tumor cell lines [6]. PHA-533533 is also a 1*H*-pyrazol-3-yl-amide derivative that counteracts tumor cell proliferation of various cell lines by inhibition of cyclin-dependent kinases (CDK) [7].

Danusertib, formely know as PHA-739358, features the 1*H*-pyrazol-3-yl-amide scaffold included in a pyrrolopyrazole template and is currently in Phase II of clinical studies, mostly for the treatment of leukemias [8]. The structure activity relationship analysis of several pyrrolopyrazole Aurora kinases inhibitors resulted in the synthesis of PHA-680632, which showed high anticancer activity on a wide range of cancer cell lines [9]. The 3-aminopyrazole moiety was incorporated in a variety of bicyclic heterocycles, the 3-amino-1*H*-thieno[2,3-c]pyrazole-5-carboxylic acid derivatives emerging as potent kinase inhibitors able to block cell cycle and tumor cell proliferation [10]. Using the same strategy, some phenylpyrazolodiazepin-7-one derivatives were prepared as conformationally rigid analoguess of aminopyrazole amide scaffold and proved to produce potent antiproliferative effects on cancer cells as selective Raf kinases inhibitors [11].

Roscovitine is a pan-selective CDKs inhibitor with multiple effects on cell proliferation, cell cycle progression and/or induction of apoptosis in cancer cells. By incorporating the 3-aminopyrazole scaffold, pyrazolo[1,5-a]-1,3,5-triazine [12] and pyrazolo[4,3-d]pyrimidine [13] derivatives were designed and synthesized as roscovitine bioisosters and demonstrated to be potent cyclin-dependent kinases inhibitors with antiproliferative activity.

**Figure 1:**
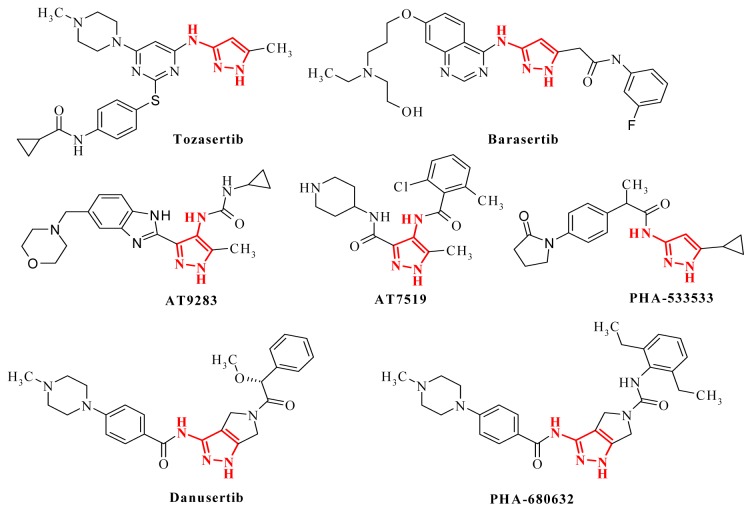
The structures of representative protein kinases inhibitors based on the aminopyrazole scaffold.

The structures of the aforementioned pyrazole derivatives with potent effects on various protein kinases are presented in Figure 1 and represent the starting point of this research.

In previous studies [14,15], we have synthesized several pyrazole derivatives. Based on our results and on literature data, in this study we designed and synthesized new aminopyrazole compounds as potential kinase inhibitors as anticancer agents. The new structures were designed using as template important bioactive scaffolds derived from the analysis of several anticancer agents, and constructed by joining aminopyrazole with thiourea and phenyl moieties.

The thiourea and acyl groups are used for their ability to form hydrogen bonds with the kinase, the 4-substituted-phenyl is designed to bond to the enzyme’s hydrophobic pocket and the 3-amino- and 5-aminopyrazole scaffold is a powerful ligand for the kinase’s ATP pocket. The thioamide is an isostere of the amide and has the advantage of being a better hydrogen bond donor and the sulfur is a superior donor for π– π* interactions, such bonds being very important in the ligand-kinase interaction [16]. In the same manner, the thiourea group works homologously to urea, the urea fragment being extensively used in the kinases inhibitors structures, such as AT9283, PHA-680632 and SNS-032. SNS-032 is a potent and selective inhibitor of CDK2, CDK7 and CDK9, and features the acylthiourea group included in a 2-aminothiazole heterocycle and the piperidine ring functions similar to the phenyl [17].

The great pharmacological potential of the aminopyrazole derivatives have prompted large-scale research aimed at developing specific synthetic routes to these compounds, the most important being the reactions of β-ketonitriles, malononitrile, alkylidenemalononitriles and their derivatives with hydrazines [18,19].

**Figure 2:**
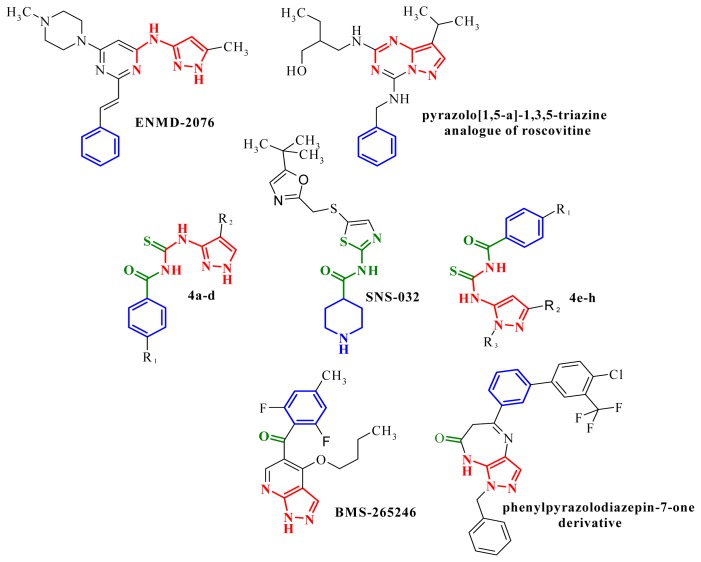
The structural design of the new compounds **4a**–**h** based on the aminopyrazole, thiourea and phenyl scaffolds.

The strategy used for the rational design of the new structures is highlighted in Figure 2 and can be observed by comparing the new aminopyrazoles **4a**–**d** and **4e**–**h** with well-known kinases inhibitors like ENMD-2076 [3], roscovitine derivatives with a pyrazolo[1,5-a]-1,3,5-triazine structure [12], SNS-032 [17], BMS-265246, a potent and selective CDK1/2 inhibitor [20], and a phenylpyrazolodiazepin-7-one derivative [11].

Alternative methods using plants and invertebrates are commonly used to determine the toxicity of newly synthesized compounds. The *Artemia salina* (brine shrimp) and *Daphnia magna* (water flea) bioassays are two invertebrate models used in studies of ecotoxicology [21,22] and to assess the general toxicology of natural compounds [23,24] and various pharmaceuticals [25], including antitumor agents [26]. These tests offer significant advantages such as speed, simplicity, low cost and good correlation with the acute toxicity observed in rodents, such as mice and rats [27,28].

A quick, economical and relevant bioassay used for the assessment of the cytotoxic activity is the phytotoxicity test, with the most widely used being the seedling growth studies. The inhibition of the root length and the modification of the cytological parameters is a simple, yet efficient method to evaluate new potential anticancer agents [29]. Additionally, the phytobiological tests can provide useful information about the genotoxicity of the new compounds [30].

In the view of the therapeutical potential of the aminopyrazoles, we prepared a series of new pyrazole derivatives and evaluated their toxicological profile using *Artemia salina*, *Daphnia magna* and their cytotoxic effects on *Triticum aestivum* roots.

## 2. Results and Discussion

### 2.1. Chemistry

**Figure 3:**
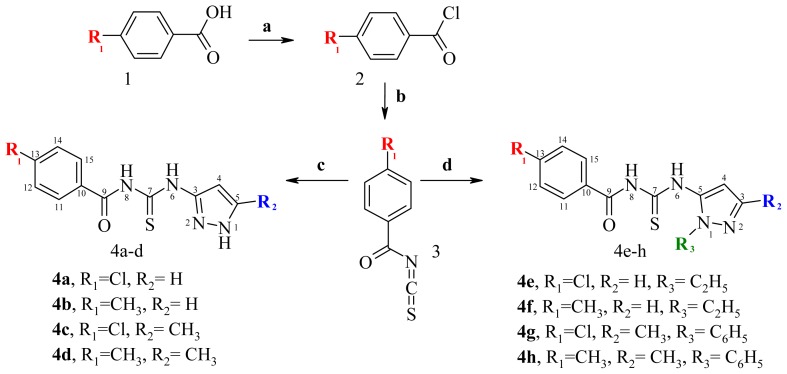
Synthetic route to new pyrazole amines derivatives. Reagents: (**a**) SOCl_2_; (**b**) NH_4_SCN; (**c**) R_2_–C_3_H_2_N_2_–NH_2_; (**d**) R_2_R_3_–C_3_HN_2_–NH_2_.

The new compounds were synthesized by the general method outlined in Figure 3, starting from benzoic substituted acids (**1**) that were converted into the corresponding benzoyl chloride (**2**) using thionyl chloride as chlorination reagent. Treating the benzoyl chlorides with ammonium isothiocyanate afforded 4-R_1_-benzoyl isothiocyanate (**3**). This was converted into the target thiourea compounds (**4a**–**h**) by reaction with various substituted pyrazole amines.

The structures of the newly synthesized compounds were confirmed on the basis of their IR, and NMR spectroscopic analysis as well as elemental analytical data.

### 2.2. Biological Screening

#### 2.2.1. Acute Toxicity Assay against *Artemia salina*

Assay results are shown in Table 1 and indicate that all compounds show a lower cytotoxicity against brine shrimp nauplii in comparison with colchicine. For the 3-aminopyrazole derivatives, the nature of the R_1_ group seems to have little influence on the toxic effect, whereas for the 5-aminopyrazole series, the 4-chlorobenzoyl derivatives are nearly 2–3 times more toxic than the corresponding 4-methylbenzoyl derivatives.

#### 2.2.2. Acute Toxicity Assay against *Daphnia magna*

The results of *D. magna* bioassay are summarized in Table 2. The toxicity of the new compounds is relatively equivalent to that of colchicine and phenazone, indicating a possible nonspecific toxic mechanism.

#### 2.2.3. Cytotoxicity Assay against *Triticum aestivum*

All the newly synthesized compounds, except **4b**, have a cytotoxic effect equivalent with that of the phenazone. All the 4-methylbenzoyl derivatives have a better cytotoxic effect than the corresponding 4-chlorobenzoyl derivatives, comparing with the *Artemia* assay, where the effect of the benzoyl R_1_ substitution is inversely. The compound **4b** presents the highest cytotoxic effect, approximately 33% of that produced by colchicine, an established antitumor drug. The results are summarized in Table 3.

### 2.3. Data Analysis

**Figure 4:**
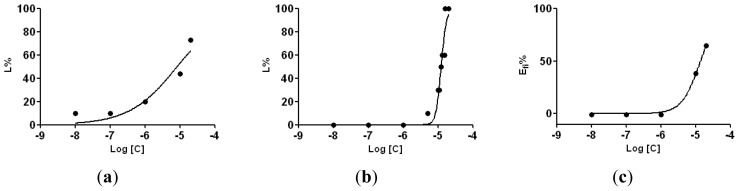
Dose-mortality curves for cytotoxic activity on *A. salina* (**a**), *D. magna* (**b**) and *T. aestivum* (**c**), exposed to compound **4e**.

For all the compounds **4a**–**h**, as well colchicine and phenazone, logarithm of dose *versus* mortality for *A. salina* and *D. magna* and logarithm of dose *versus* inhibitory effect on the embryonic roots of *T. aestivum* were determined and plotted, as presented for the compound **4e** in the Figure 4.

**Figure 5:**
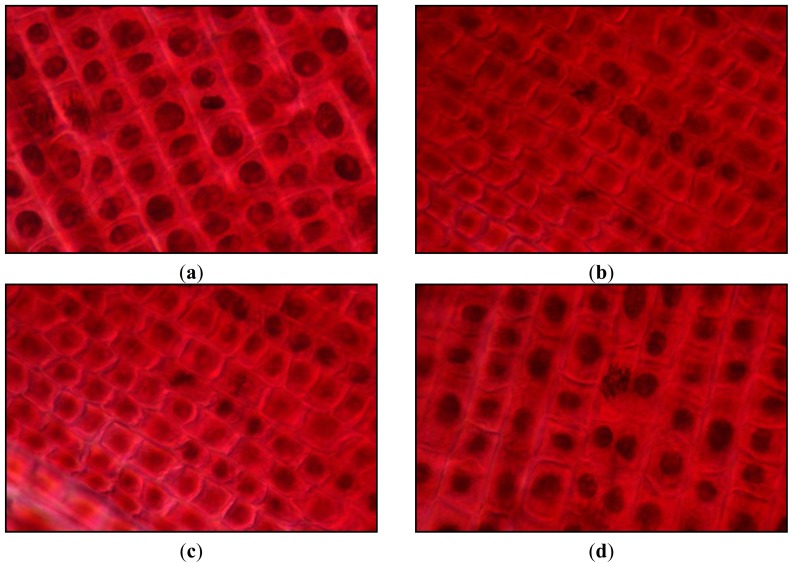
Kariokinetic film modifications observed in *Triticum* test: (**a**) polyploid telophase induced by colchicine at 0.1 μmol/L; (**b**) metaphase in tropokinesis and anaphase with late chromosomes induced by **4b** at 1 μmol/L; (**c**) metaphase in tropokinesis induced by **4g** at 0.1 μmol/L; (**d**) disorganised metaphase induced by **4a** at 10 μmol/L (ob. 40×).

Microscopic examination revealed that, at the highest concentration, colchicine presented a total cytotoxic effect, no mitotic divisions being observed and at the rest of the concentrations was toxic for cell division leading to kariokinetic film modifications: hypertrophied nuclei with abnormal shapes, metaphases and anaphases in tropokinesis, disorganised metaphases, polyploid telophases (Figure 5a). At the highest concentration, phenazone induced rare abnormal cell divisions such as metaphases and anaphases in tropokinesis, disorganised metaphases; at concentrations from 0.01 to 10 μmol/L, no kariokinetic film modifications were observed.

Compounds **4b**, **4c**, **4d**, **4f**, **4g** and **4h** at concentrations of 10 and 20 μmol/L, induced rare abnormal cell divisions: metaphases and anaphases in tropokinesis (Figure 5b,c), anaphases with late chromosomes (Figure 5b) and disorganized metaphases indicating a potential antitumor activity. At concentrations between 0.01 and 1 μmol/L, no modifications were observed.

Compound **4a** induced at all tested concentrations kariokinetic film modifications: metaphases, anaphases in tropokinesis and disorganised metaphases (Figure 5d), an effect similar to that observed in colchicine at concentrations under 10 μmol/L, but without polyploid divisions.

## 3. Experimental Section

### 3.1. Chemistry

#### 3.1.1. General

All starting materials, reagents, and solvents were purchased from commercial suppliers. All melting points were measured in open capillary tubes on an IA9100 (Electrothermal, UK). The NMR spectra were recorded on a Gemini 300 BB instrument (Varian, Palo Alto, CA, USA) at room temperature, operating at 300 MHz for ^1^H and 75.075 MHz for ^13^C. The chemical shifts were recorded as δ values in ppm units downfield of tetrametylsilane used as internal standard. The coupling constants values are reported in hertz and the splitting patterns are abbreviated as follows: s, singlet; d, doublet; t, triplet; q, quartet and b, broad. The IR spectra were recorded on a FT/IR-4200 spectrometer (JASCO, Tokyo, Japan) with an ATR PRO450-S accesory. The elemental analyses were performed on a PerkinElmer 2400 Series II CHNS/O Elemental Analyzer (Shelton, CT, USA).

#### 3.1.2. Synthetic Procedures

A solution of 4-R_1_-benzoic acid (0.1 mol) in anhydrous 1,2-dichlorethane is refluxed with thionyl chloride (14.5 mL, 0.2 mol) until evolution of gas is completed. The solvent and the excess thionyl chloride are removed by reduced pressure distillation. The raw obtained 4-R_1_-benzoyl chloride (10 mmol) is dissolved in anhydrous acetone (15 mL), added to a solution of ammonium thiocyanate (10 mmol) in acetone (15 mL) and refluxed for one hour. The ammonium chloride is removed by filtration and the suitable pyrazole amine (10 mmol) is added. The mixture is stirred for two to three hours and then poured into ten times its volume of cold water. *N*-(4-R_1_-benzoyl)-*N*′-(1*H*-pyrazolyl)- thiourea derivatives (**4a**–**h**) precipitated as solids. The compounds were recrystallized from ethanol or isopropanol.

##### *N*-(4-chlorobenzoyl)-*N*′-(1*H*-pyrazol-3-yl)-thiourea (**4a**)

Yield 76%, mp 180–182 °C. IR (cm^−1^): 3179 (N–H); 3128 (N–H); 3047 (N–H); 1667 (C=O); 1521 (C–N). ^1^H NMR (DMSO-d_6_, ppm): 13.10 (s, NH, H-1); 12.80 (bs, NH, H-6); 11.75 (bs, NH, H-8); 7.99 (d, *J* = 8.5 Hz, 2H, H-11, H-15); 7.73 (d, *J* = 2.2 Hz, 1H, H-5); 7.60 (d, *J* = 8.5 Hz, 2H, H-12, H-14); 7.08 (d, *J* = 2.2 Hz, 1H, H-4).^13^C NMR (DMSO-d_6_, ppm): 176.80 (C=S); 167.74 (C=O); 146.80 (C-5); 138.06 (C-3); 138.05 (C-13); 130.97 (C-10); 130.73 (C-11, C-15); 128.52 (C-12, C-14); 98.12 (C-4). Calcd. for C_11_H_9_ClN_4_OS: C, 47.06; H, 3.23; N, 19.96; S, 11.42. Found: C, 47.34; H, 3.19; N, 20.12; S, 11.26%.

##### *N*-(1*H*-pyrazol-3-yl)-*N*′-(4-methylbenzoyl)-thiourea (**4b**)

Yield 63%, mp 169–171 °C. IR (cm^−1^): 3178 (N–H); 3124 (N–H); 3055 (N–H); 1659 (C=O); 1527 (C-N). ^1^H NMR (DMSO-d_6_, ppm): 13.22 (s, NH, H-1); 12.80 (bs, NH, H-6); 11.49 (s, NH, H-8); 7.89 (d, *J* = 8.2 Hz, 2H, H-11, H-15); 7.72 (d, *J* = 2.3 Hz, 1H, H-5); 7.34 (d, *J* = 8.2 Hz, 2H, H-12, H-14); 7.07 (d, *J* = 2.3 Hz, 1H, H-4); 2.38 (s, 3H, CH_3_). ^13^C NMR (DMSO-d_6_, ppm): 177.01 (C=S); 168.56 (C=O); 146.73 (C-5); 143.78 (C-13); 138.55 (C-3); 129.07 (C-11, C-15); 128.95 (C-10); 128.82 (C-12, C-14); 98.14 (C-4); 21.16 (CH_3_). Calcd. for C_12_H_12_N_4_OS: C, 57.37; H, 4.65; N, 21.52; S, 12.32. Found: C, 55.22; H, 4.59; N, 21.66; S, 12.24%.

##### *N*-(4-chlorobenzoyl)-*N*′-(5-methyl-1*H*-pyrazol-3-yl)-thiourea (**4c**)

Yield 66%, mp 205–206 °C. IR (cm^−1^): 3222 (N–H); 3112 (N–H); 3042 (N–H); 1666 (C=O); 1525 (C–N). ^1^H NMR (DMSO-d_6_, ppm): 13.01 (s, NH, H-1); 12.47 (s, NH, H-6); 11.65 (s, NH, H-8); 7.98 (d, *J* = 8.6 Hz, 2H, H-11, H-15); 7.59 (d, *J* = 8.6 Hz, 2H, H-12, H-14); 6.87 (s, 1H, H-4); 2.25 (s, 3H, CH_3_). ^13^C NMR (DMSO-d_6_, ppm): 176.41 (C=S); 167.68 (C=O); 146.89 (C-5); 138.39 (C-3); 138.05 (C-13); 130.98 (C-10); 130.74 (C-11, C-15); 128.52 (C-12, C-14); 97.39 (C-4); 10.77 (CH_3_). Calcd. for C_12_H_11_ClN_4_OS: C, 48.90; H, 3.76; N, 19.01; S, 10.88. Found: C, 48.77; H, 3.88; N, 19.29; S, 10.80%.

##### *N*-(5-methyl-1*H*-pyrazol-3-yl)-*N*′-(4-methylbenzoyl)-thiourea (**4d**)

Yield 65%, mp 208–211 °C. IR (cm^−1^): 3268 (N–H); 3162 (N–H); 3059 (N–H); 1663 (C=O); 1527 (C-N). ^1^H NMR (DMSO-d_6_, ppm): 13.13 (s, NH, H-1); 12.51 (s, NH, H-6); 11.42 (s, NH, H-8); 7.89 (d, *J* = 7.5 Hz, 2H, H-11, H-15); 7.33 (d, *J* = 7.5 Hz, 2H, H-12, H-14); 6.86 (s, 1H, H-4); 2.38 (s, 3H, CH_3_); 2.24 (s, 3H, CH_3_). ^13^C NMR (DMSO-d_6_, ppm): 176.71 (C=S); 168.55 (C=O); 146.84 (C-5); 143.78 (C-13); 138.61(C-3); 129.00 (C-10); 129.10 (C-11, C-15); 128.84 (C-12, C-14); 97.47 (C-4); 21.20 (CH_3_); 10.82 (CH_3_). Calcd. for C_13_H_14_N_4_OS: C, 56.92; H, 5.14; N, 20.42; S, 11.69. Found: C, 57.00; H, 5.14; N, 20.64; S, 11.52%.

##### *N*-(4-chlorobenzoyl)-*N*′-(1-ethyl-1*H*-pyrazol-5-yl)-thiourea (**4e**)

Yield 70%, mp 160–161 °C. IR (cm^−1^): 3379 (N–H); 3175 (N–H); 1661 (C=O); 1526 (C–N). ^1^H NMR (DMSO-d_6_, ppm): 12.07 (s, NH, H-6); 11.76 (s, NH, H-8); 8.00 (d, *J* = 8.7 Hz, 2H, H-11, H-15); 7.62 (d, *J* = 8.7 Hz, 2H, H-12, H-14); 7.45 (d, *J* =1.9 Hz, 1H, H-3); 6.42 (d, *J* = 1.9 Hz, 1H, H-4); 4.02 (q, *J* = 7.1 Hz, 2H, CH_2_); 1.35 (t, *J* = 7.1 Hz, 3H, CH_3_). ^13^C NMR (DMSO-d_6_, ppm): 180.74 (C=S); 167.28 (C=O); 138.18 (C-13); 137.62 (C-3); 135.65 (C-5); 130.86 (C-10); 130.83 (C-11, C-15); 129.17 (C-12, C-14); 101.41 (C-4); 42.96 (CH_2_); 14.75 (CH_3_). Calcd. for C_13_H_13_ClN_4_OS: C, 50.57; H, 4.24; N, 18.14; S, 10.38. Found: C, 50.51; H, 4.19; N, 18.22; S, 10.44%.

##### *N*-(1-ethyl-1*H*-pyrazol-5-yl)-*N*′-(4-methylbenzoyl)-thiourea (**4f**)

Yield 69%, mp 172–173 °C. IR (cm^−1^): 3379 (N–H); 3167 (N–H); 1659 (C=O); 1530 (C–N). ^1^H NMR (DMSO-d_6_, ppm): 12.15 (s, NH, H-6); 11.84 (s, NH, H-8); 7.92 (d, *J* = 8.3 Hz, 2H, H-11, H-15); 7.45 (d, *J* = 2.1 Hz, 1H, H-3); 7.35 (d, *J* = 8.3 Hz, 2H, H-12, H-14); 6.44 (d, *J* = 2.1 Hz, 1H, H-4); 4.02 (q, *J* = 7.3 Hz, 2H, CH_2_); 2.39 (s, 3H, CH_3_); 1.35 (t, *J* = 7.3 Hz, 3H, CH_3_). ^13^C NMR (DMSO-d_6_, ppm): 180.81 (C=S); 168.15 (C=O); 143.92 (C-13); 137.63 (C-3); 135.67 (C-5); 129.13 (C-11, C-15); 129.10 (C-10); 128.99 (C-12, C-14); 101.37 (C-4); 43.01 (CH_2_); 21.23 (CH_3_); 14.77 (CH_3_). Calcd. for C_14_H_16_N_4_OS: C, 58.31; H, 5.59; N, 19.43; S, 11.12. Found: C, 58.09; H, 5.65; N, 19.60; S, 10.99%.

##### *N*-(4-chlorobenzoyl)-*N*′-(3-methyl-1-phenyl-1*H*-pyrazol-5-yl)-thiourea (**4g**)

Yield 73%, mp 176–178 °C. IR (cm^−1^): 3266 (N–H); 3170 (N–H); 1671 (C=O); 1553 (C–N). ^1^H NMR (DMSO-d_6_, ppm): 12.40 (s, NH, H-6); 11.75 (s, NH, H-8); 7.84 (d, *J* = 8.5 Hz, 2H, H-11, H-15); 7.50 (d, *J* = 8.5 Hz, 2H, H-12, H-14); 7.48 (d, *J* = 7.4 Hz, 2H, C_6_H_5_); 7.41 (t, *J* = 7.4 Hz, 2H, C_6_H_5_); 7.30 (t, *J* = 7.4 Hz, 1H, C_6_H_5_); 6.50 (s, 1H, H-4); 2.18 (s, 3H, CH_3_). ^13^C NMR (DMSO-d_6_, ppm): 180.05 (C=S); 167.01 (C=O); 147.85 (C-5); 138.23 (C-3); 138.20 (C-13); 136.73 (C_6_H_5_); 130.76 (2C, C_6_H_5_); 130.59 (C-10); 129.21 (C-12, C-14); 128.55 (C-11, C-15); 127.56 (C_6_H_5_); 123.84 (2C, C_6_H_5_); 102.80 (C-4); 13.86 (CH_3_). Calcd. for C_18_H_15_ClN_4_OS: C, 58.30; H, 4.08; N, 15.11; S, 8.65. Found: C, 58.24; H, 3.99; N, 15.19; S, 8.59%.

##### *N*-(3-methyl-1-phenyl-1*H*-pyrazol-5-yl)-*N*′-(4-methylbenzoyl)-thiourea (**4h**)

Yield 68%, mp 202–203 °C. IR (cm^−1^): 3277 (N–H); 3155 (N–H); 1666 (C=O); 1548 (C-N). ^1^H NMR (DMSO-d_6_, ppm): 12.51 (s, NH, H-6); 11.68 (s, NH, H-8); 7.83 (d, *J* = 8.1 Hz, 2H, H-11, H-15); 7.55 (d, *J* = 7.5 Hz, 2H, H-17, H-21); 7.48 (t, *J* = 7.5 Hz, 2H, C_6_H_5_); 7.37 (t, *J* = 7.5 Hz, 1H, C_6_H_5_); 7.31 (d, *J* = 8.1 Hz, 2H, H-12, H-14); 6.57 (s, 1H, H-4); 2.37 (s, 3H, CH_3_); 2.25 (s, 3H, CH_3_). ^13^C NMR (DMSO-d_6_, ppm): 180.43 (C=S); 167.95 (C=O); 147.90 (C-5); 143.97 (C-13); 138.27 (C-3); 136.82 (C_6_H_5_); 129.25 (2C, C_6_H_5_); 129.12 (C-12, C-14); 128.92 (C-11, C-15); 128.80 (C-10); 127.63 (C_6_H_5_); 123.90 (2C, C_6_H_5_); 102.88 (C-4); 21.20 (CH_3_); 13.90 (CH_3_). Calcd. for C_19_H_18_N_4_OS: C, 65.12; H, 5.18; N, 15.99; S, 9.15. Found: C, 64.99; H, 5.22; N, 15.90; S, 9.09%.

### 3.2. Biological Screening

Colchicine (COL) and phenazone (PHZ) were used as positive control in all determinations, using the same molar concentrations as the tested compounds. Colchicine is a toxic, antitumor alkaloid that binds to tubulin and prevents its polymerization into microtubules, thereby blocking formation of the mitotic spindle and arresting nuclear division at metaphase [31]. Phenazone, known also as antipyrine, is an analgesic and antipyretic with no significant cytotoxic effect and was chosen as control because of the similar pyrazole structure [32]. The general toxicity of the new compounds was evaluated using *Artemia salina* and *Daphnia magna*. The analysis of the compound’s effect on *Triticum aestivum* embryonic root length and of the karyokinetic film’s modifications was performed in order to evaluate the new aminopyrazoles anticancer potential.

#### 3.2.1. Acute Toxicity Assay against *Artemia salina*

Lethality toward *A. salina* was determined using procedures described in the literature [21,33] with some modifications. Brine shrimp eggs were obtained from a local aquarium shop (Bucharest, Romania) and hatched in breakers containing artificial sea water (40 g/L salinity) at 25 ± 1 °C for 48 h in a plant growth chamber (Sanyo MLR-351 H, San Diego, CA, USA) in the dark and under continuous aeration. The newly hatched nauplii were separated from the shells, transferred to fresh sea water with a micropipette. Assays were performed in 9-well culture plates (Labsystems, Vantaa, Finland). Each well contained 10 larvae in 500 μL final volume of each dilution of the new compounds. The final DMSO concentration was 1% (*v*/*v*) and compound concentrations were in the range of 0.01–20 μmol/L (0.01, 0.1, 01, 10 and 20 μmol/L). Artificial sea water and 1% solvent control were used as negative control. Tests were performed in quadruplicate. Due to the absence of specific information about the stability in the presence of light of the pyrazole derivatives, the bioassay was performed under the same conditions as those used for hatching. After 24 h, the number of survivors was counted and recorded. Larvae were considered dead only if they did not move their appendages for 10 s during observation.

#### 3.2.2. Acute Toxicity Assay against *Daphnia magna*

*D. magna* test was performed according to the method described in literature [34]. Cytotoxicity testing was performed in 15 mL glass tubes, using 10 daphnids/tube. The compounds were tested using the same concentrations as for the *Artemia* test. The bioassay was then repeated at the concentrations ranging from 1 to 20 μmol/L (1, 5, 10, 11, 12, 13, 15 and 16 μmol/L) in order to determine LC_50_ for each compound, including the positive control. Lethality was recorded after 24 h, considering dead the organisms that did not move their appendages for 30 s. All experiments were conducted in the dark, in a plant growth chamber (Sanyo MLR-351 H, San Diego, CA, USA) at 25 ± 1 °C.

#### 3.2.3. Acute Toxicity Assay against *Triticum aestivum*

The assessment of plant cell cytotoxicity was carried out by determining the maximal dilution of the extractive solutions which, depending on the time of action, influences the radicular elongation and the karyokinetic film [35,36].

*Triticum aestivum* (Boema cultivar), supplied by SC Adaflor SRL (Tulcea, Romania) was selected as the test plant. Dry caryopses were soaked for 24 h in distilled water and allowed to germinate on moist filter paper until the main radicle attained 1 cm. Ten caryopses with 1 cm long embryonic roots were placed in Petri dishes of 90 mm diameter, for 24 h in contact with 15 mL of the tested compounds at the same concentrations as those used in *A. salina* test. The bioassay was conducted at 25 ± 1 °C, 75% relative humidity and in the absence of light, in a plant growth chamber (Sanyo MLR-351 H, San Diego, CA, USA). A negative control sample was prepared with distilled water. The length of the embryonic root was measured and any modifications of the karyokinetic film were observed after staining the primary wheat root meristems with diluted acetic orcein solution, using a Labophot 2 Nikon microscope (ocular 10×, ob. 40×, 100×) (Nikon, Chiyoda-ku, Tokyo, Japan).

### 3.3. Data Analysis

For the *A. salina* and *D. magna* bioassays, the mortality percentage was plotted against the logarithm of concentrations. The compounds concentration that kills 50% of the larvae (LC_50_) and their 95% confidence limit were calculated from the dose-response equation determined by the least squares fit method, using the GraphPad Prism version 5.0 (SanDiego, CA, USA, 2007) software.

For the *Triticum* bioassay the inhibitory effect of the tested solutions was calculated with Excel 2003, using the formula described in our previous paper [35]. The inhibitory effect was plotted against the logarithm of concentrations and the concentration inhibiting the root elongation with 50%, compared with the negative control, (IC_50_) was calculated as described for *A. salina* and *D. magna*.

For each regression analysis, the goodness of fit (r^2^) and confidence intervals (α = 0.05) were calculated using GraphPad Prism version 5.0 software.

## 4. Conclusions

We designed a series of new compounds as potential protein kinases inhibitors by interlocking aminopyrazole, thiourea and phenyl moieties. The new 3-aminopyrazole and 5-aminopyrazole derivatives were synthesized using easy and accessible methods, and their structures confirmed by IR and NMR spectroscopic analysis and elemental analytical data.

The compounds were tested on *Artemia salina* and *Daphnia magna* to assess their acute toxicity and on *Triticum aestivum* in order to estimate their antitumor potential. The compound *N*-(1*H*-pyrazol-3-yl)-*N*′-(4-methylbenzoyl)-thiourea (**4b**) displayed the best cytotoxic effect, correlated with a low general toxicity, and could represent the lead molecule for new anticancer agents based on the aminopyrazole structure.

## Figures and Tables

**Table 1:** The toxic activity of compounds **4a**–**h** in the brine shrimp lethality bioassay.

Compound	LC_50_ (μmol/L)	LC_50_ 95% confidence interval (μmol/L)	Goodness of fit (*r*^2^)
**4a**	4.68	– a	0.7103
**4b**	4.61	0.87–24.43	0.9057
**4c**	8.15	2.37–27.99	0.9110
**4d**	7.85	–a	0.6080
**4e**	3.84	–a	0.6175
**4f**	8.83	–a	0.7304
**4g**	3.05	0.35–26.18	0.8336
**4h**	11.09	–a	0.7348
COL	2.17	0.64–7.38	0.9490
PHZ	6.64	–a	0.7518

a – 95% confidence interval is very wide and could not be calculated.

**Table 2:** The toxic activity of compounds **4a**–**h** in the *D. magna* bioassay.

Compound	LC_50_ (μmol/L)	LC_50_ 95% confidence interval (μmol/L)	Goodness of fit (*r*^2^)
**4a**	9.44	8.59–10.38	0.9839
**4b**	15.14	–a	0.9973
**4c**	12.16	11.35–13.06	0.9452
**4d**	13.65	12.82–14.49	0.9904
**4e**	12.91	12.59–13.21	0.9858
**4f**	13.34	12.68–14.03	0.9483
**4g**	15.07	–a	0.9459
**4h**	13.40	12.74–14.13	0.9634
COL	13.90	13.37–14.45	0.9702
PHZ	15.24	14.42–16.07	0.9442

a – 95% confidence interval is very wide and could not be calculated.

**Table 3:** The phytotoxicity of the compounds **4a**–**h** in the *Triticum* bioassay.

Compound	IC_50_ (μmol/L)	IC_50_ 95% confidence interval (μmol/L)	Goodness of fit (*r*^2^)
**4a**	9.82	5.70–16.87	0.9793
**4b**	2.91	0.16–53.58	0.7685
**4c**	13.61	12.39–14.96	0.9980
**4d**	11.75	7.52–18.32	0.9596
**4e**	14.35	10.54–19.54	0.9921
**4f**	11.19	9.20–13.61	0.9921
**4g**	13.46	10.64–17.06	0.9694
**4h**	10.79	10.21–11.40	0.9995
COL	0.95	0.12–7.31	0.9020
PHZ	13.24	8.83–19.86	0.9482
